# Polyethylene glycol 3350 plus electrolytes for pediatric chronic constipation: An open‐label clinical study in Japan

**DOI:** 10.1111/ped.14102

**Published:** 2020-04-27

**Authors:** Mayuko Gondo, Satoru Nagata, Kazuhiko Shinbo, Akira Oota, Takeshi Tomomasa

**Affiliations:** ^1^ Department of Pediatrics Tokyo Women’s Medical University Tokyo Japan; ^2^ Clinical Development Department EA Pharma Co., Ltd. Tokyo Japan; ^3^ Pal Children’s Clinic Gunma Japan

**Keywords:** Constipation, Japan, paediatrics, polyethylene glycol 3350, spontaneous bowel movement

## Abstract

**Background:**

Despite the abundance of study evidence for its efficacy and tolerability for the treatment of constipation in other countries, polyethylene glycol 3350 plus electrolytes (PEG3350+E) was not available in Japan until recently. The purpose of this study was to establish the efficacy and safety of PEG3350+E for the treatment of functional constipation in children in Japan.

**Methods:**

Japanese children aged 2–14 years with a mean spontaneous bowel movement (SBM) frequency of 2 times/week or less for at least 2 months prior to informed consent were enrolled into the study. After a 2‐week screening period, treatment with PEG3350+E was initiated on the day of enrollment and continued for 12 weeks. Change in SBM frequency from screening period week 2 (baseline) to treatment period week 2 was set as the primary endpoint. Secondary endpoints and adverse events were also examined.

**Results:**

Thirty‐nine patients were enrolled and completed the 12‐week study period. The SBM frequency (mean ± SD) at baseline and treatment period week 2 was 1.00 ± 0.89 and 6.54 ± 4.38, respectively. The change in SBM frequency was 5.54 ± 4.55 (one‐sample *t* test, *P* < 0.0001) and remained stable through week 12. Stool consistency was also improved over the entire treatment period. Three mild adverse drug reactions were reported: decreased appetite, abdominal pain, and diarrhea (each in 1 of 39 [2.6%] patients).

**Conclusion:**

PEG3350+E can be considered as a new treatment option for chronic constipation in children in Japan. Clinical trial registration number: Japic CTI‐163167.

Constipation is accompanied by unpleasant symptoms such as difficulty in defecation, sensation of incomplete evacuation, abdominal pain due to decreased frequency of bowel movements (BMs), decreased amount of each defecation, and hard stool. Systematic reviews of chronic functional constipation (constipation without organic disease) in children outside of Japan showed that the prevalence (BM < 3 times/week) in children aged 0–18 years ranged from 0.7% to 29.6% and there was no clear difference between the sexes.^1^, ^2^ In Japan, however, there have been few reports on the prevalence of chronic functional constipation in children. In a survey on BM frequency in 6917 elementary school students in Hiroshima city, 18.5% (male, 13.2%; female, 24.1%) of children had BMs < 2 or 3 times per week.^3^

Constipation is managed by an improvement in diet and lifestyle, self‐treatment using over‐the‐counter drugs, and treatment, mainly with drugs, at medical institutions. Pharmacotherapy at medical institutions in Japan includes bulk cathartics, such as carmellose sodium and dioctyl sodium sulfosuccinate plus casanthranol, stimulant cathartics, such as sennosides A and B and sodium picosulfate hydrate, and salt cathartics, such as magnesium oxide and magnesium sulfate. Salt cathartics and stimulant cathartics are the most frequently used agents in Japan. However, salt cathartics may result in loose stool due to excessive secretion of water and hypermagnesemia, while stimulant cathartics may become addictive and cause secondary failure if used long term, leading to low satisfaction with these drugs. Hence, the identification of drugs with high efficacy that are suitable for long‐term use is warranted.

In countries other than Japan, polyethylene glycol (PEG) cathartics are used as standard therapy for chronic constipation in adults and children. There is good evidence for the efficacy and safety of PEG 3350 plus electrolytes (PEG3350+E) in the treatment of constipation; PEG3350+E has been shown to be more effective than lactulose, a disaccharide cathartic, or placebo.^4^, ^5^ Treatment with PEG3350+E has also been described in the National Institute for Health and Care Excellence (NICE, UK) guidelines (2010) and the European Society for Paediatric Gastroenterology Hepatology and Nutrition and the North American Society for Pediatric Gastroenterology, Hepatology and Nutrition guidelines (2014).^6^, ^7^ In Japan, there is reference to PEG in the Practice Guidelines for Chronic Functional Constipation in Children,^8^ but until recently, it was not available in this indication. Of note, PEG is used as an intestinal tract cleaning agent for endoscopy of the large bowel.

Since PEG3350+E is widely used globally, and evidence for its efficacy and safety has accumulated, an open‐label Japanese clinical study was designed based on the dosage regimens used in other countries, to evaluate the efficacy and safety of PEG3350+E given orally for 12 weeks following a 2‐week screening period in paediatric patients with chronic constipation.

## Methods

### Study design

This open‐label, non‐randomized, phase 3 study was conducted between October 2016 and June 2017 (from the first informed consent to the last observation) at 11 medical institutions located in Japan. After a 2‐week screening period, treatment with PEG3350+E was initiated on the day of enrollment and continued for 12 weeks.

### Patients

Japanese children and adolescents aged 2–14 years old with a mean spontaneous BM (SBM) frequency of 2 times/week or less for at least 2 months prior to informed consent were enrolled in this study. For inclusion, patients had to have at least one of the following symptoms related to SBM: straining during at least 25% of defecations, scybalum or hard stool in at least 25% of defecations, anal hemorrhage during at least 25% of defecations, and pain during at least 25% of defecations. This study included not only patients with functional constipation but also patients with constipation‐predominant irritable bowel syndrome (IBS‐C). In view of the symptomatology and pathophysiology of the condition,^9^, ^10^ latent class analysis suggests that functional constipation and IBS‐C differ mostly in severity rather than in the type of symptoms.^11^

Written informed consent was obtained from the legal representative (parent) of the patient; however, written assent was also obtained from patients who were of junior high school age at the time of informed consent and, whenever possible, from patients who were of elementary school age and younger.

Patients were excluded from the study if they had organic constipation with past or current history of gastrointestinal obstruction, Hirschsprung's disease, past or current history of intestinal hernia; symptomatic constipation; drug‐induced constipation; complications with megacolon or encopresis; severe reflux esophagitis; slow transit colon constipation; or excretory disorder constipation.

After providing consent, patients underwent vital sign measurements (pulse, blood pressure, weight) to confirm their eligibility and were provisionally enrolled 15 days prior to the first day of treatment. Patients with not more than 4 SBMs during the 2‐week screening period and without loose stool (Bristol Stool Form Scale [BSFS] type 6 and type 7^12^) were eligible and enrolled.

### Treatment

PEG3350+E (6.9 g sachet, powder for oral solution; Norgine Limited, Uxbridge, UK) is a powder formulation in sachets containing 6.5625 g PEG3350, 0.1754 g sodium chloride, 0.0893 g sodium bicarbonate, and 0.0251 g potassium chloride per sachet. Each sachet was reconstituted in approximately 62.5 mL water.

The dosage of PEG3350+E was regulated depending on the patient’s age and the dose adjustment criteria (Table 1). The patient’s legal representative (parent) checked the patient’s SBM status and adjusted the dose of PEG3350+E based on the criteria. PEG3350+E was administered once or twice daily based on dosage (twice‐daily administration was given in the morning and evening). For patients requiring a dose increase, the dose was increased every other day. Rescue medications such as sodium picosulfate hydrate, bisacodyl, or glycerin enema were allowed only for patients who experienced no BM for at least 72 consecutive hours between the start of the run‐in period and the last observation.

**Table 1:** Dose adjustment criteria based on Bristol Stool Form Scale

Age (years)	Initial dosage	Bristol Stool Form Scale on the day before administration				Upper limit
	Sachets/day	No BM, type 1, type 2	Type 3, type 4	Type 5	Type 6, type 7	Sachets/day
2–6	1	Plus 1	Unchanged†	Minus 1	Minus 2 or suspended	4
7–11	2	Plus 1	Unchanged†	Minus 1	Minus 2 or suspended	4
12–14	2	Plus 2	Unchanged	Unchanged	Minus 2 or suspended	6

^†^ Increase in dosage was allowed in the presence of pain on defecation and/or anal hemorrhage even when the Bristol Stool Form Scale was 3. BM, bowel movement.

### Study assessments

After enrollment, patients received the study treatment from day 1 to day 84. To evaluate efficacy, patients’ electronic diaries were used to investigate the date and time of BMs, stool consistency, sensation of incomplete evacuation (for patients with the ability to confirm a feeling of complete evacuation), date and time of rescue medication use, and number of study treatment sachets used in the morning and evening. Stool consistency was self‐assessed by the legal representative (parent) of the patient on a scale from type 1 (hard lumps) to type 7 (liquid consistency) according to the BSFS. Patients visited the hospital at weeks 0, 2, 4, 8, and 12. Vital signs were also assessed at each visit. Standard laboratory tests for hematology, biochemistry, and urinalysis were performed at baseline and week 12 or at the time of discontinuation.

### Study endpoints

The primary endpoint was the change in SBM frequency from screening period week 2 (baseline) to treatment period week 2. Secondary endpoints included the following changes from baseline to each treatment period week: (i) SBM and complete SBM (CSBM) frequency (CSBM was defined as an SBM with a sense of complete evacuation); (ii) SBM and CSBM responder rates (defined as 3 or more SBMs/CSBMs per week and an increase of at least 1 SBM/CSBM per week compared with baseline); (iii) number of days to the first SBM and CSBM; (iv) stool consistency, as measured by the BSFS; (v) use of rescue medication; and (vi) number of sachets of PEG3350+E taken.

### Safety analysis

The following were summarized for safety evaluation: coding of adverse events (AEs), incidence of AEs, and clinical laboratory tests and vital signs. Categorization of AEs was based on the Medical Dictionary for Regulatory Activities version 19.0.

### Ethics

The ethical, scientific, medical, and pharmacological appropriateness of this clinical study was reviewed and approved by the Institutional Review Boards of Yokohama Minoru Clinic, Goshozuka Clinic, Saiseikai Yokohamashi Tobu Hospital, Saitama City Hospital, National Center for Child Health and Development, Tokyo Medical University Hospital, Tokyo Women’s Medical University Hospital, Juntendo University Hospital, and Hiroshima City Hiroshima Citizens Hospital. This clinical study was conducted in compliance with the ethical principles based on the Declaration of Helsinki, the Law on Securing Quality, Efficacy and Safety of Products Including Pharmaceuticals and Medical Devices, and Good Clinical Practice guidelines.

### Statistical analysis

Unless otherwise specified, the level of significance was two‐sided at 5%, and the confidence interval (CI) was two‐sided at 95%. At the week of discontinuation or last visit, if a patient had < 5 days of diary entries regarding defecation during a week, that week was considered not assessable and was treated as a missing value. All data were analyzed using SAS, version 9.3 (SAS Institute Inc., Cary, NC, USA) by AC Medical Co., Ltd. (Tokyo, Japan). Since this was a pediatric study and obtaining informed consent was expected to be difficult for multiple collections of blood samples, among other factors, the sample size of 35 was selected as a practicable number of patients.

## Results

### Study patients

**Figure 1:**
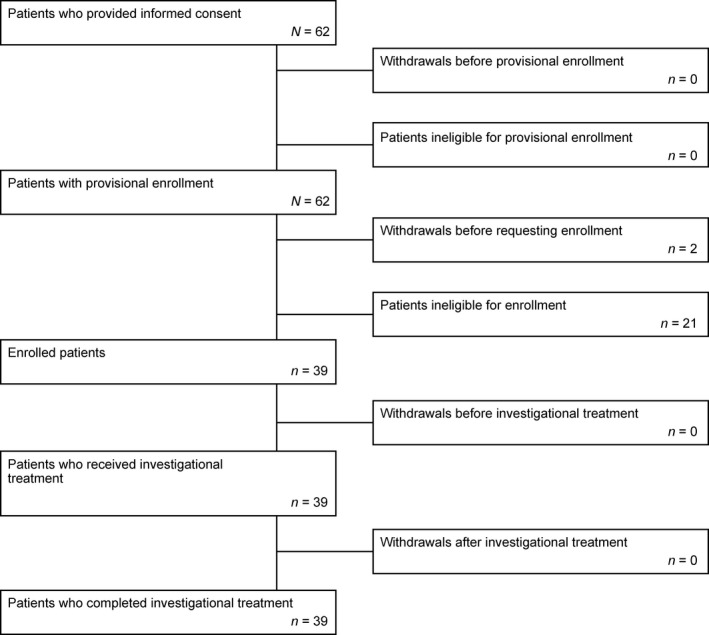
Patient disposition.

Of the 62 patients who provided consent, no patient discontinued before provisional enrollment or was judged ineligible for provisional enrollment. Of these 62 patients, two discontinued before enrollment and 21 were judged ineligible for enrollment. Patients with an SBM frequency of more than 4 times in the 2‐week run‐in period (*n* = 17), patients who had used rescue medication within 72 h of a BM during the 2‐week run‐in period (*n* = 2), and patients who were considered ineligible by the investigator or sub‐investigator due to any other reasons (*n* = 2) were ineligible for inclusion. Thus, 39 patients were enrolled and completed 12 weeks of study treatment (Fig. 1). Patient demographics and baseline characteristics of the enrolled patients are shown in Table 2. Overall, 20 patients had the ability to confirm a feeling of complete evacuation and they were assessed for the CSBM‐related endpoints.

**Table 2:** Patient demographics and baseline characteristics

Characteristic	*N* = 39
Female sex	23 (59.0)
Age, (years)	4.9 ± 3.1
2–6	32 (82.1)
7–11	4 (10.3)
12–14	3 (7.7)
Ability to confirm feeling of complete evacuation	20 (51.3)
Fulfilled criteria for IBS‐C	6 (15.4)
SBMs per week†	1.00 ± 0.89
CSBMs per week†	0.95 ± 0.76
Use of rescue medication†	29 (74.4)
Stool consistency score‡	2.43 ± 1.04

Data are mean ± SD or *n* (%).

^†^ Baseline value was based on week 2 of the screening period.

^‡^ Stool consistency was assessed using the Bristol Stool Form Scale. CSBM, complete spontaneous bowel movement; IBS‐C, constipation‐predominant irritable bowel syndrome; SBM, spontaneous bowel movement; SD, standard deviation.

### Analysis of primary endpoint

**Figure 2:**
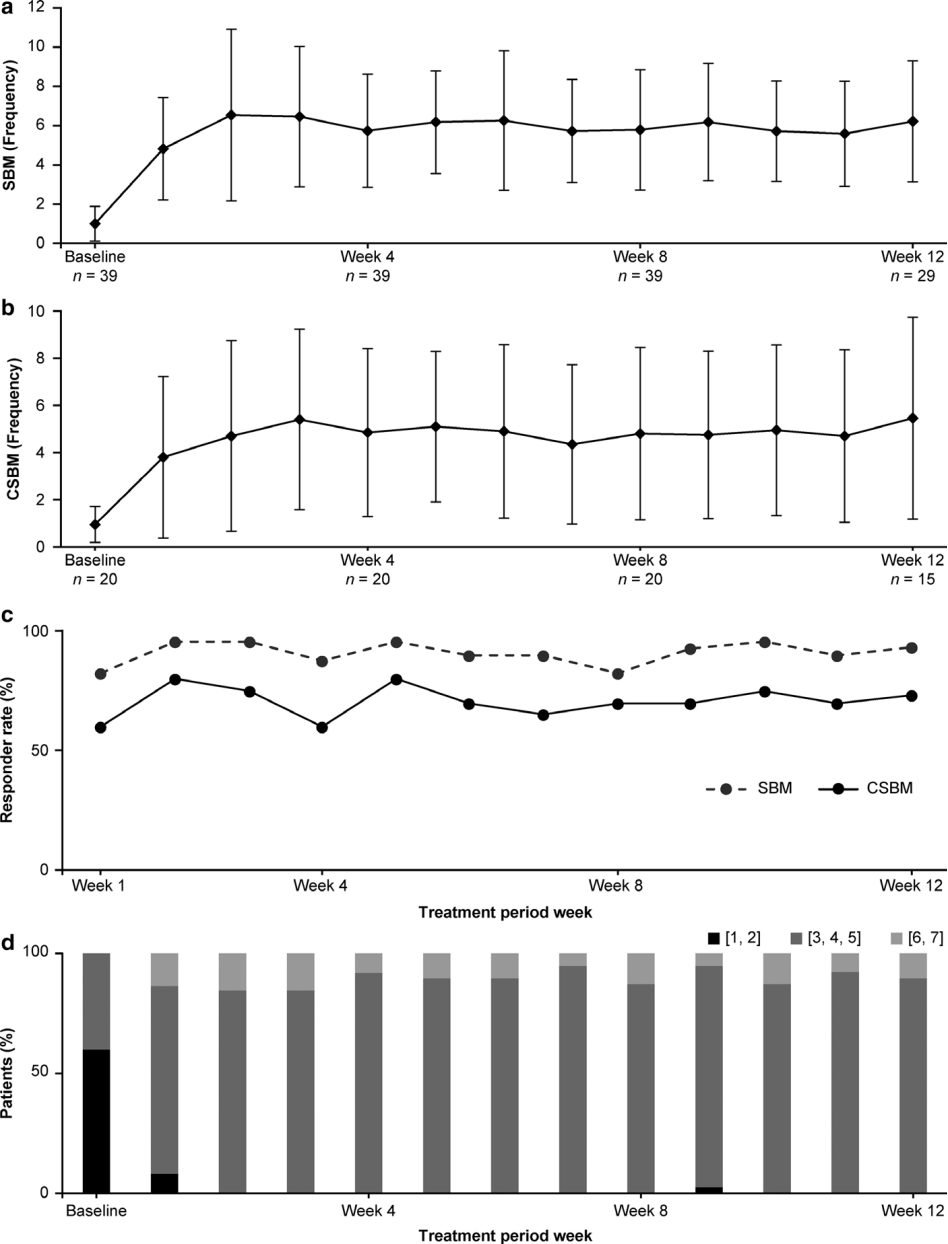
Polyethylene glycol 3350 plus electrolytes (PEG3350+E) in the treatment period. (a) spontaneous bowel movement (SBM) frequency during each week of the treatment period; (b) complete spontaneous bowel movement (CSBM) frequency during each week of the treatment period; (c) SBM and CSBM responder rates during each week of the treatment period; (d) ratio of type of stool consistency (median) categorized using the Bristol Stool Form Scale [1, 2], [3, 4, 5], and [6, 7]. SBM and CSBM responder rates were defined as 3 or more SBMs/CSBMs per week and an increase of at least 1 SBM/CSBM per week compared with baseline.

The SBM frequency (mean ± SD) at baseline and treatment period week 2 was 1.00 ± 0.89 and 6.54 ± 4.38, respectively. The change in SBM frequency during the same period was 5.54 ± 4.55, representing a significant increase (one‐sample *t*‐test, *P* < 0.0001). The SBM frequencies in each treatment period week in the modified intent‐to‐treat set are shown in Fig. 2a.

### Analysis of secondary endpoints

#### Changes in SBM frequency and CSBM frequency from baseline to each treatment period week

The SBM frequency is shown in Fig. 2a. The changes in SBM frequency from baseline to each treatment period week were 3.82 ± 2.48, 5.54 ± 4.55, 4.74 ± 3.07, 4.79 ± 3.16, and 5.18 ± 3.13 at treatment weeks 1, 2, 4, 8, and 12, respectively; the SBM frequencies in treatment week 1 and thereafter were significantly increased compared to those at baseline (one‐sample *t*‐test, *P* < 0.0001).

The CSBM frequency is shown in Fig. 2b. The changes in CSBM frequency in the modified intent‐to‐treat set from baseline to each treatment period week were 2.85 ± 3.07, 3.75 ± 3.91, 3.90 ± 3.46, 3.85 ± 3.54, and 4.46 ± 4.05 at treatment weeks 1, 2, 4, 8, and 12, respectively. The CSBM frequencies in treatment week 1 and thereafter were significantly increased compared to those at baseline (one‐sample *t*‐test, *P* < 0.001).

Ten out of 39 patients had < 5 days of diary entries regarding defecation during week 12; therefore, week 12 was considered not assessable and was treated as a missing value for these patients. As a result, the number of patients at week 12 was lower than the number of patients at the other time points studied, with 29 patients in the SBM assessment (Fig. 2a) and 15 patients in the CSBM assessment (Fig. 2b) at week 12.

#### SBM and CSBM responder rates for each treatment period week

#### SBM responder rates for each treatment period week in the modified intent‐to‐treat set are shown in Fig. 2c. SBM responder rates were 82.1% at treatment week 1 (95% CI: 67.3–91.0) and remained stable through week 12, ranging from 82.1% to 94.9%. Those of CSBM for each treatment period week in the modified intent‐to‐treat set are shown in Fig. 2c.

#### Number of days to the first SBM and CSBM

The median number of days to the first SBM and first CSBM in the modified intent‐to‐treat set estimated by the Kaplan–Meier method was 2.0 days (95% CI: 2.0–3.0) and 3.0 days (95% CI: 1.0–6.0), respectively.

#### Stool consistency as measured by BSFS

For patients in the modified intent‐to‐treat set with BMs in each treatment period, the median weekly BSFS score according to the seven categories was defined as the assessment scale value. The proportions of patients in each period when the median was classified as [1, 2], [3, 4, 5], or [6, 7] are shown in Fig. 2d. The median weekly stool consistency scores (mean ± SD) as measured by BSFS were 2.4 ± 1.0 at baseline and 4.1 ± 1.2, 4.5 ± 0.8, 4.4 ± 0.6, 4.4 ± 0.8, and 4.4 ± 0.7 in treatment period weeks 1, 2, 4, 8, and 12, respectively. From treatment period week 2 through week 12, this measurement ranged from 4.2 to 4.5, showing that the stool consistency consistently improved.

#### Use of rescue medication

**Figure 3:**
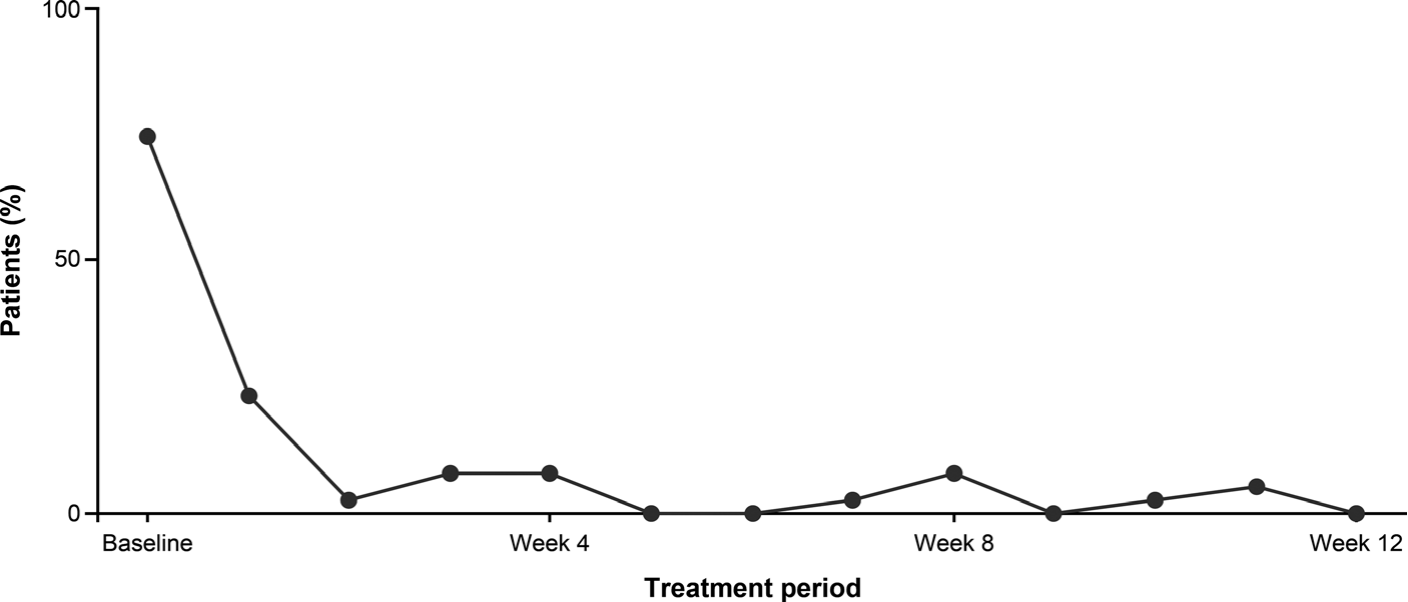
Rescue medication use in the treatment period. Status of rescue medication use in the modified intent‐to‐treat set at baseline and during each week of polyethylene glycol 3350 plus electrolytes (PEG3350+E) treatment.

The use of rescue medication in the modified intent‐to‐treat set at baseline and during each week of the treatment period is shown in Fig. 3. The proportions of patients who used rescue medication were 74.4% at baseline and 23.1%, 2.6%, 7.7%, 7.7%, and 0.0% in treatment weeks 1, 2, 4, 8, and 12, respectively. Thus, the proportions were reduced from treatment period week 1, then remained stable from treatment period week 2 through week 12.

#### Number of sachets of PEG3350+E used

**Figure 4:**
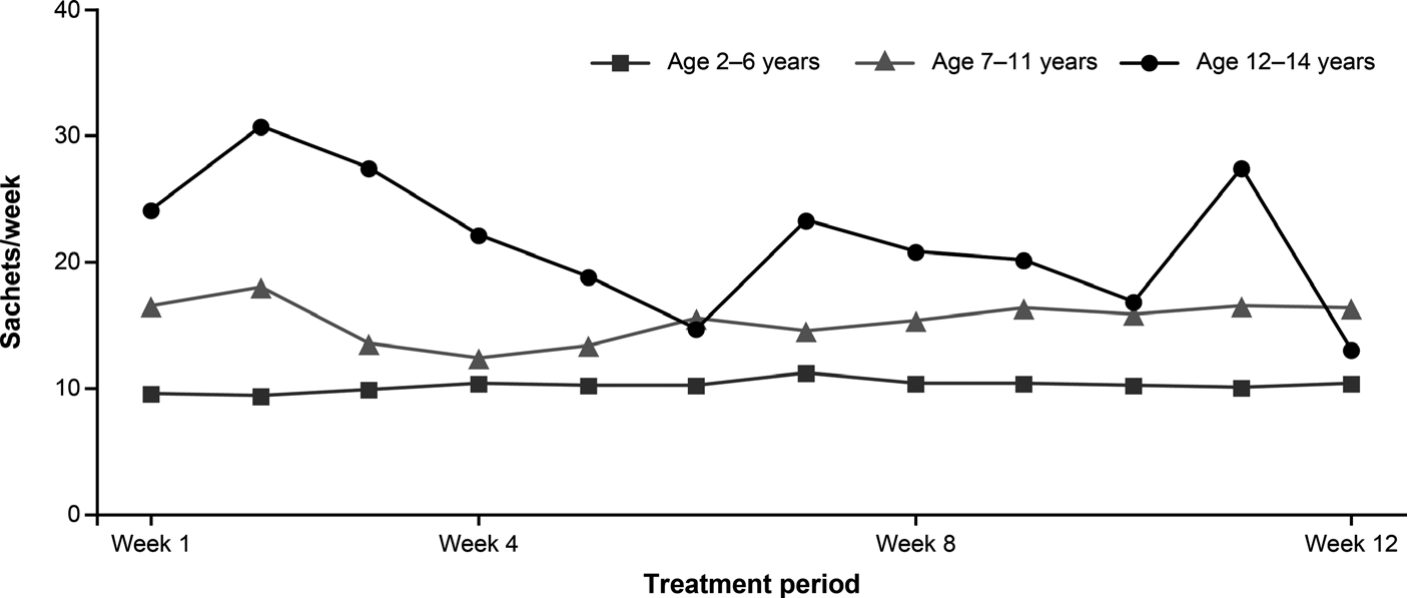
PEG3350+E, polyethylene glycol 3350 plus electrolytes (PEG3350+E) use in the treatment period. Summary statistics for the mean number of sachets of PEG3350+E taken during each week of the treatment period by age group (2–6, 7–11, and 12–14 years).

The summary statistics for the number of sachets of PEG3350+E taken in each treatment period week by age group (2–6, 7–11, or 12–14 years) are shown in Fig. 4. In patients aged 2–6 years, the mean ± SD number of sachets taken was 9.5 ± 3.4 in treatment period week 1, 9.4 ± 6.2 in week 2, 10.3 ± 6.9 in week 4, 10.4 ± 7.9 in week 8, and 10.3 ± 7.1 in week 12. In patients aged 7–11 years, the mean ± SD number of sachets taken was 16.5 ± 6.1 in treatment period week 1, 18.0 ± 8.8 in week 2, 12.3 ± 3.5 in week 4, 15.3 ± 4.6 in week 8, and 16.3 ± 5.3 in week 12. In patients aged 12–14 years, the mean ± SD number of sachets taken was 24.0 ± 6.0 in treatment period week 1, 30.7 ± 8.3 in week 2, 22.0 ± 7.2 in week 4, 20.7 ± 6.4 in week 8, and 13.0 ± 15.6 in week 12.

### Safety analysis

Table 3 provides a summary of the AEs observed. The incidence of AEs was 74.4% (29/39 patients). The incidence of adverse drug reactions (ADRs) was 7.7% (3/39 patients). No deaths, serious AEs other than death, severe AEs, or AEs leading to treatment discontinuation were reported. AEs occurring in ≥ 2 patients were upper respiratory tract inflammation (25.6%, 10/39 patients), influenza and urticaria (10.3% each, 4/39 patients), gastroenteritis and nasopharyngitis (7.7% each, 3/39 patients), and conjunctivitis and eczema (5.1% each, 2/39 patients). The following three events, all of which were mild in severity, were classified as ADRs: decreased appetite, abdominal pain, and diarrhea (2.6% each, 1/39 patients). Of these ADRs, no event occurred in ≥ 2 patients. No clinically significant changes were observed in laboratory tests and vital signs.

**Table 3:** Summary of adverse events

	Patients (*N* = 39) *n* (%)
Adverse events	29 (74.4)
Adverse drug reactions (related to PEG3350+E)	3 (7.7)
Death	0 (0.0)
Serious adverse events (except those leading to death)	0 (0.0)
Adverse events leading to discontinuation	0 (0.0)
Severe adverse events	0 (0.0)

PEG3350+E, polyethylene glycol 3350 plus electrolytes.

## Discussion

This study showed a satisfactory effect of PEG3350+E on chronic constipation in children. 

We conducted baseline‐controlled, open‐label, multicenter trials among Japanese children with chronic constipation in order to study the safety and effectiveness of PEG3350+E. After the observation period, we administered PEG3350+E for 12 weeks in patients with not more than 4 SBMs during the 2‐week screening period. Adherence to treatment was good, as this laxative is easy to take because it is readily dissolved in water; additionally, there were no restrictions on the timing of the dose, and the dosage could be adjusted. As a result, the frequency of SBMs and CSBMs increased, along with improvements in the fecal properties from the first week of PEG3350+E administration compared with baseline. The effect lasted throughout the administration period without being attenuated. Tolerability was also good, with most cases indicating a steady shift without use of rescue medication. Approximately 25% of children with chronic functional constipation continue to experience constipation as adults, with a high rate of recurrence; however, the prognosis is said to be improved by early diagnosis and treatment.^13^ Constipation in adults is known to be a factor for colon cancer,^14^ mental disorders such as depression and anxiety,^15^ and myocardial infarction.^16^

The general principle of treatment is to start by identifying the presence of a fecal plug, which is a large fecal mass, and if one does exist, consider removal by a medical specialist. This is because medical treatment has almost no effect where a fecal plug is present.^6^, ^7^, ^17^ Lifestyle guidance, diet therapy, and drug therapy are provided if there is no fecal plug. High‐fiber foods such as vegetables and seaweed are recommended for diet therapy.^18^ For drug therapy, it is recommended to start with an osmotic laxative,^7^, ^19^ using a stimulant laxative as rescue medication in the event the osmotic laxative is ineffective.^17^ However, guidelines on medical care for chronic functional constipation in children recommend that lifestyle guidance, diet therapy, and drug therapy should be provided before medical treatment. If these treatments do not work, the possible presence of a fecal plug should be considered, and specialist medical treatment may be introduced. This approach is considered more realistic for patients in Japan.

The Japanese guidelines also include a general principle of starting with an osmotic laxative, such as a saline purgative or a saccharide purgative, for maintenance therapy for chronic functional constipation in children.^8^ Osmotic laxatives mainly affect the descending colon, sigmoid colon, and rectum, absorbing water within the gastrointestinal tract, increasing the volume of intestinal contents, and facilitating defecation. The main effects are to soften stools and to reduce pain when defecating. Maltose and lactulose are often used during infancy, while lactulose and magnesium hydroxide are often used from childhood onwards. Until now, the only drugs that have been used in Japan, and which are covered by insurance in the country, are lactulose, sodium picosulfate hydrate, and bisacodyl. The latter two drugs are not considered first‐line treatments because they are stimulant laxatives. While the Japanese guidelines on constipation in children state that osmotic laxatives are effective against chronic constipation and recommend their use, lactulose is the only drug for children that is covered by insurance.

Few osmotic laxatives have been studied in placebo‐controlled trials. On the other hand, trials have been carried out with PEG, with results reporting its usefulness.^20^ The NICE (UK) guidelines recommend using PEG3350+E as a first‐line treatment for removing fecal mass and as maintenance therapy for constipation in children (< 18years).^6^ It is recommended that diet and lifestyle modification be used in combination with a laxative, not a laxative alone, as a first‐line treatment. Both the European and North American Societies for Paediatric Gastroenterology Hepatology and Nutrition recommend PEG (with or without electrolytes) for use as a first‐line treatment for removing fecal mass and as maintenance therapy in children with constipation.^7^ The high efficacy of PEG as a treatment for constipation in children has been reported in many studies.^21^, ^22^ A meta‐analysis also reported greater usefulness of PEG versus lactulose.^20^

PEG3350 can bind approximately 100 H_2_O molecules per one molecule via a hydrogen bond; therefore, orally administered PEG3350 dissolved in water is only absorbed to a small extent and allows water to reach the colon without being decomposed.^23^, ^24^, ^25^ Smooth defecation can be expected using PEG3350+E since it delivers water to the intestines, softens and enlarges stool, and increases the peristaltic action of the small and large intestines via the effect of the osmotic pressure of PEG.^23^

While previous Asian studies have assessed PEG4000 without electrolytes for chronic functional constipation in children,^26^, ^27^ this is the first Asian study to assess the efficacy of PEG3350+E for chronic functional constipation in children. Comparison of this study with other European studies is difficult because of the differences in patient characteristics and definition of stool frequency, among other factors.^4^, ^5^, ^28^ We compared our results with those of the European studies; all the studies adjusted the treatment dose based on the patients’ stool consistency and showed an improvement in BM with a low incidence of ADRs. Hardikar *et al* reported that PEG3350+E significantly improved not only the frequency of defecation and stool consistency but also constipation‐related symptoms, such as abdominal pain, rectal bleeding, pain on defecation, straining, and soiling in toilet‐trained children.^28^ We did not assess such symptoms as efficacy endpoints, but the safety results showed that abdominal pain (*n* = 1) was the only constipation‐related AE. This is the first report to assess SBM and CSBM responder rates with PEG3350+E for pediatric chronic constipation. These parameters clearly show the efficacy of PEG3350+E for the treatment of constipation.

This study demonstrated the safety and efficacy of PEG3350+E for the treatment of chronic functional constipation in children in Japan, using the same dosage that has been used in Europe.

Recently, it was reported that PEG3350+E significantly resolved constipation compared with placebo in the 2‐week, randomized, double‐blinded confirmatory phase of a PEG3350+E study, and the efficacy of PEG3350+E lasted for the entire 52‐week extension phase, with good tolerability in Japanese adults with chronic constipation.^29^

## Conclusion

Despite being widely used in other countries for several years, PEG formulations have only recently been approved for the treatment of constipation in Japan; however, being the first PEG formulation in Japan, PEG3350+E is considered a new treatment option for chronic constipation in children aged 2 years or older.

## Disclosure

Takeshi Tomomasa has served as a clinical advisor for this study (EA Pharma Co., Ltd.). Kazuhiko Shinbo and Akira Oota are employees of EA Pharma Co., Ltd. Satoru Nagata has received lecture fees from EA Pharma Co., Ltd. Satoru Nagata and Mayuko Gondo have received a research grant from EA Pharma Co., Ltd.

## Author contributions

K.S., A.O., and T.T. contributed to the conception and planning of the study; S.N. collected the data; M.G., S.N., and T.T. contributed to interpretation of the data; and M.G. and S.N. drafted the manuscript. All authors read and approved the final manuscript.
